# Congenital Ptosis Associated With Adduction as a Dysinnervation Disorder: A Report of a Rare Case

**DOI:** 10.7759/cureus.40422

**Published:** 2023-06-14

**Authors:** Ghadah Alnosair, Hassan Alhashim, Mustafa Alhamoud, Hissah Alturki

**Affiliations:** 1 Pediatric Ophthalmology and Strabismus, Dammam Medical Complex, Dammam, SAU; 2 Ophthalmology, Imam Abdulrahman Bin Faisal University, Dammam, SAU; 3 Ophthalmology, King Fahd Hospital of the University, Khobar, SAU; 4 Ophthalmology, Dhahran Eye Specialist Hospital, Dhahran, SAU

**Keywords:** blepharoptosis, dysinnervation disorders, congenital ptosis, pediatric ophthalmology, congenital dysinnervation

## Abstract

The levator palpebrae superioris is the primary muscle responsible for elevation of the eyelid. This muscle is innervated by the third intracranial nerve. Any pathology affecting the muscle or the supplying nerve can lead to blepharoptosis. In this study, we share our experience of a two-year-old baby boy patient who presented with a rare congenital disorder manifested as blepharoptosis increased with adduction bilaterally with no limitation of ocular muscles action except bilateral underaction of inferior oblique muscles. To our knowledge, this unusual presentation has not been previously reported in the literature. We aim in this report to build more knowledge on such a rare clinical presentation. Based on the findings, this could be a case of congenital innervation dysgenesis syndrome (CID)/congenital cranial dysinnervation disorders (CCDDs). CCDDs/CID is a group of conditions that includes blepharoptosis as part of their clinical presentation. This group of conditions includes Duane's retraction syndrome, congenital fibrosis of extraocular muscles, and monocular elevation defect.

## Introduction

Blepharoptosis is defined as an abnormally inferior displacement of the upper eyelid margin in the primary position. Moreover, when the upper eyelid extends 3 mm or more from the center of the pupil, it is defined as blepharoptosis. It can be categorized according to the onset of the disease, or the laterality, congenital or acquired, and unilateral and bilateral involvement, respectively. It could be further classified based on the cause into neurogenic, aponeurotic, myogenic, mechanical, and traumatic. Most common cases of congenital blepharoptosis are attributed to myogenic causes in which there was maldevelopment of levator palpebrae superioris muscle, while acquired cases are mostly attributed to aponeurotic in which there was some sort of damage to levator aponeurosis caused by excessive stretching [1].

The levator palpebrae superioris muscle is responsible primarily for raising the upper eyelid and maintaining its position. It receives its innervation from the superior branch of the oculomotor nerve which is the third cranial nerve (CN III). This nerve originates from the midbrain from a single caudal subnucleus located within the oculomotor nucleus. Moreover, the innervation of both levator palpebrae superioris muscles of the right and left eyes originates from a single subnucleus. Therefore, any single lesion could result in bilateral ptosis [2,3]. On the other hand, the medial rectus muscle is responsible primarily for adducting the eye. It receives its innervation from the inferior branch of the oculomotor nerve (CN III) which originated from the medial rectus subnucleus within the oculomotor nucleus to supply the ipsilateral medial rectus [4,5]. The symptoms of congenital oculomotor nerve (CN III) palsy include ptosis and the inability to raise, lower, or adduct the globe. Another possibility is dilated pupils. Ptosis is very rarely found alone. This palsy may be partial or complete. In congenital CN III palsies, aberrant innervation is not frequently observed. In aberrant regeneration of the oculomotor nerve there will be change in ptosis with movement of the extraocular muscles (EOMs) [1].

A couple of congenital dysinnervation disorders to EOMs have been described before in the literature such as Duane's retraction syndrome, congenital fibrosis of EOMs, and monocular elevation defects which could be grouped under congenital innervation dysgenesis syndrome (CID)/congenital cranial dysinnervation disorders (CCDDs) which are a group of congenital, non-progressive disorders and all share the same general pathophysiology of having a neurological disturbance and have a wide spectrum of presentation that affects the eye movements either horizontally or vertically that might be associated with blepharoptosis resulting in various types of strabismus and abnormal head positions and sometimes extends to have non-ophthalmologic associations [6,7]. Aberrant innervation of the ocular and facial muscles results in CCDDs. This condition is thought to be the result of genetic mutations related to the development of cranial motor neurons. This condition might affect one or multiple cranial nerve nuclei or their axonal connections [8]. In this case report, we aim to share our experience in a rarely encountered clinical presentation of congenital bilateral blepharoptosis increased with right and left eye adductions.

## Case presentation

**Figure 1:**
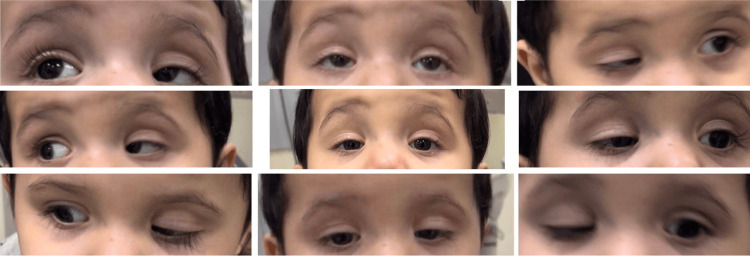
Nine gazes of the extraocular muscle motility showing full extraocular muscle movements.

A two-year-old full-term healthy boy without medical history and born by normal vaginal delivery presented to the pediatric ophthalmology clinic at Dammam Medical Complex Hospital in Dammam, Saudi Arabia with a chief complaint of abnormal lid movement noticed by the mother since birth. There was no history of red eye, excessive lacrimation or discharges, squint, head trauma, recent infections, neurological diseases, and other systemic or ocular symptoms. There was no family history of squints or childhood eye diseases. Upon examination, the visual acuity assessment for his age was central, steady, and maintained in both eyes with no fixation preference, IOP was difficult to measure due to the patient's age but digitally was within the normal range, the pupil was normal (round, regular, reactive, symmetrical, and no apparent pupillary defect), there was normal eyelid crease, and the EOM motility was within full range bilaterally (Figure 1).

**Figure 2:**
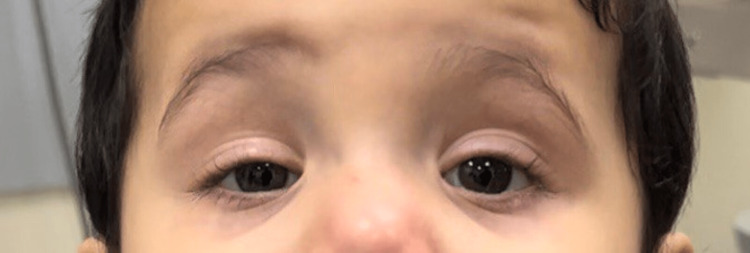
Primary position picture showing bilateral ptosis not covering the visual axis with the use of the frontalis muscle.

Our patient has a chin-up position. In the primary position, the patient had a bilateral inferior displacement of the upper eyelid with MRD 1 measuring 3mm not blocking the visual axis, but the patient uses his frontalis muscle to elevate his eyelid as shown in Figure 2.

This ptosis increases adduction in the right and left gaze obstructing the visual axis with a widening of palpebral fissure on abduction. There was no change in the eyelid position with jaw movements. For other anterior segment evaluations, it was unremarkable, and for dilated fundus examination, it shows normal optic disc and macula bilaterally. His cycloplegic refraction assessment shows +4.00 -1.25X180 in the right eye (OD) and +3.50 -1.00X180° in the left eye (OS). MRI was done looking for brainstem abnormalities and showed bilateral almost symmetrical peri trigonal high T2 signal intensity which is separated from the lateral ventricle by a normal white matter cleavage which is a normal finding. Since the condition showed no effect on the patient's vision and no amblyopia was observed, the patient was managed by glasses, observation, and referral to oculoplastic for consultation. 

## Discussion

Blepharoptosis is classified into congenital or acquired. Acquired blepharoptosis might be mechanical that occurs when the eyelid is too heavy to be raised by the muscles such as in blepharochalasis, orbital fat prolapses, and eyelid tumors. Moreover, acquired blepharoptosis is trauma that affects the levator palpebrae superioris muscles or the oculomotor nerve. However, in our case, acquired causes were essentially ruled out because the patient presented with bilateral blepharoptosis in association with adduction since birth and there was no history of trauma or mechanical injury [1]. The oculomotor nerve supplies the levator palpebrae superioris muscles of both eyes and isolated pathology involving the oculomotor nerve will only explain blepharoptosis, not the association with adduction as in our case [4,5].

In previous literature, Duane's retraction syndrome, congenital fibrosis of EOMs, monocular elevation defects, and Marcus-Gunn-Jaw-winking syndrome were referred to as congenital dysinnervation disorders termed congenital innervation dysgenesis syndrome (CID)/congenital cranial dysinnervation disorders (CCDDs). Those disorders result from aberrant innervation between ocular and facial muscles. Mutations in the genes which are responsible for the development and connectivity of the cranial nerves or their axonal connections result in CCDDs. All those conditions are congenital and non-progressive and have neurological involvement. Patients affected with those congenital conditions may present with blepharoptosis, different types of strabismus, and abnormal head positions [6-8]. This is similar to the presentation of our case as the patient presented with increased bilateral blepharoptosis when adducting the eye. 

CCDDs are an umbrella term that includes many syndromes. Moebius syndrome is one of the conditions that fall under CCDDs. In Moebius syndrome, facial and abducens nerves are involved. Patients will present with restricted lateral gaze and facial dropping involving the forehead and eyebrows. In our case, no restriction to lateral gaze or facial dropping was observed upon examination which excluded Moebius syndrome. Another condition is congenital fibrosis of EOMs which might involve restriction to the vertical gaze was also excluded. Duane's retraction syndrome in which patients present with globe retraction, narrowing of the palpebral fissure in adduction, is the closest differential diagnosis although it does not exactly fit the description of our case because there is no limitation of adduction or abduction and no globe retraction [6].

Another case similar to our case but older was reported by Mendes et al. who presented an 8-year-old child with congenital right ptosis increased on right adduction and left ptosis increased on left adduction managed only by refraction and patching for her amblyopia with no surgical intervention [5]. The difference is that the ptosis was more in the right eye causing amblyopia in that patient. Otherwise, no other cases were reported under the name of ptosis on adduction in the literature. The definitive diagnosis for such a case is not defined and could represent a disorder under the umbrella of CCDs.

One of the differential diagnoses is pseudoptosis. In pseudoptosis, the upper eyelid may appear inferiorly displaced due to its position with respect to the eye, and the underlying cause is not the eyelid itself but due to other causes. Causes of pseudoptosis include enophthalmos in which the upper eyelid is not supported posteriorly leading to ptosis. Other causes are hypotropia in which the ptosis disappears when the patient fixates with the affected eye, lid retraction in the contralateral eye that makes the other eye appear falsely ptotic, and redundant eyelid skin and orbital fat prolapse in the elderly [1]. However, our patient does not have any of the conditions that cause pseudoptosis. Moreover, on examination, there was no improvement in ptosis in one eye when we cover the other eye [9].

Another important differential diagnosis is aberrant regeneration of the oculomotor nerve which results from trauma to the oculomotor nerve or direct compression of the nerve by tumors or aneurysms. Although this condition cannot be ruled out in our case, it is still unlikely because our patient has had ptosis since birth and has no history of trauma [10].

Blepharoptosis repair can be achieved by structural correction by repositioning the defective muscles or functional correction by suspending the muscles to an adjacent structure. Most commonly used surgical options include conjunctiva-Müller’s muscle resection, Fasanella-Servat procedure, internal and external levator resection, levator aponeurosis repair, frontalis muscle suspension, and Whitnall’s ligament suspension. All those procedures aim to move the eyelid to its normal position [1]. In our case, the patient was given glasses and referred to oculoplasty for consultation although the parents are not interested in doing the surgery right now if indicated.

## Conclusions

In this study, we presented a case of a two-year-old child with congenial bilateral blepharoptosis that increases with adduction of the left and right eye. All examinations and imaging were normal except for the blepharoptosis and chin-up position. According to our findings, this case might be a CCDD. However, the possibility of aberrant regeneration of the oculomotor nerve cannot be ruled out. Because there was no effect on the vision and no amblyopia, our patient was given glasses and referred to oculoplasty for consultation. 
